# Vibrational Spectra of Zeolite Y as a Function of Ion Exchange

**DOI:** 10.3390/molecules26020342

**Published:** 2021-01-11

**Authors:** Magdalena Król, Andrzej Koleżyński, Włodzimierz Mozgawa

**Affiliations:** Faculty of Materials Science and Ceramic, AGH University of Science and Technology, 30 Mickiewicza Av., 30-059 Krakow, Poland; andrzej.kolezynski@agh.edu.pl (A.K.); mozgawa@agh.edu.pl (W.M.)

**Keywords:** zeolite, faujasite, ion exchange, cationic sites, lattice dynamics calculations, FTIR spectroscopy, Raman spectroscopy

## Abstract

Zeolite Y is one of the earliest known and most widely used synthetic zeolites. Many experimental investigations verify the valuable ion exchange capability of this zeolite. In this study, we assessed the effects of ion exchange on its vibrational spectra. We applied classical lattice dynamics methods for IR and Raman intensity calculations. Computed spectra of optimized zeolite Y structures with different cations were compared with experimental data. The spectra obtained in this study are in agreement with previous experimental and computational studies on zeolites from the faujasite group.

## 1. Introduction

Zeolites are a group of materials characterized by microporous aluminosilicate frameworks with numerous applications like, e.g., ion exchange and sorption processes or catalysis [1,2]. In the latter application, various cationic forms of faujasite play very important roles. However, due to various Si/Al ratios and the resulting local ordering, a detailed analysis of their structural properties is not an easy task. A common tool used in the structural analysis of zeolite-type materials is vibrational spectroscopy; unfortunately, due to their complex structures, a detailed and reliable analysis of the properties and shapes of vibrational spectra is a very difficult task, which usually requires additional support from theoretical calculations [3,4]. The simulation of vibrational IR and Raman spectra in a complex environment (crystal, solution) is a very challenging and widely explored topic. Molecular dynamics is, of course, the method of choice to simulate the IR spectra when a well-defined minimum is not representative of the phase space [5,6,7,8,9].

Since the unit cell size of a typical zeolite is quite big, containing hundreds (in the case of zeolite Y) or more atoms, it is still rare to find theoretical works reporting results of ab initio calculations of vibrational spectra. In the past, the usual approach was based on the assumption that, due to the hierarchical structure of typical zeolites, vibrational spectrum can be analyzed using theoretical results obtained for isolated structural elements (primary and secondary building blocks), and this approach turned out to be quite successful [10]. However, if one wants to analyze (very often) the subtle effects of an extra-framework cation exchange, the sorption processes of small molecules or processes undergoing during catalysis on structural changes and vibrational spectra properties more accurate periodic models definitely have to be used in the calculations. The problem is, however, that such models are still beyond the practical reach of quantum mechanical methods (besides the simplest cases, like, e.g., silicalite frameworks for less complicated zeolites like faujasite), and a feasible alternative is (before more powerful computers will be at our disposal) the use of classical molecular mechanics methods—the crude approximations ignoring electronic interactions but, in the case of zeolites, able to provide reasonable results.

The extra-framework cations can occupy different sites in the zeolite structure. The cation distribution in the FAU structure is usually described as follows [11]:

- sites I—located in the hexagonal prisms, which connect the so-called sodalite cages,
- sites I′—inside the sodalite cages facing site I,
- sites II—in front of the six rings inside the supercages and
- sites III and III′ or J—in the supercages near the 12-ring or four-ring windows.

Although there is considerable literature on the location of cations in faujasites [12,13,14], most of the works are based on structural analyses of real materials obtained through ion exchange. More recently, several works that dealt with Monte Carlo simulations used to describe the distribution of cations in a faujasite structure were published [15,16,17,18,19]. It was found that the method based on a Monte Carlo algorithm allows predicting both the aluminum location and cationic distribution in the material, which can be hydrated or not [15]. It is worth mentioning that the works that use computational methods and that are strictly devoted to the subject of extra-framework ions, focus rather on determining how the presence of various cations influence the adsorption capacity towards gases [16,17] or water molecules [18,19]. The modeling of theoretical vibrational spectra is, however, rare in this type of work.

In this work, we present the results of a series of calculations performed for model periodic structures of various cationic forms of zeolite Y (with a silica-to-alumina ratio 5:1) using GULP (general utility lattice program) software [20,21] and the classical molecular mechanics approach. In all cases, full geometry optimization (unit cell size and atomic positions) was done and vibrational spectra calculated. In order to confirm the validity of obtained results, samples of respective cationic forms of faujasite were synthesized and vibrational spectra measured.

## 2. Results and Discussion

**Figure 1:**
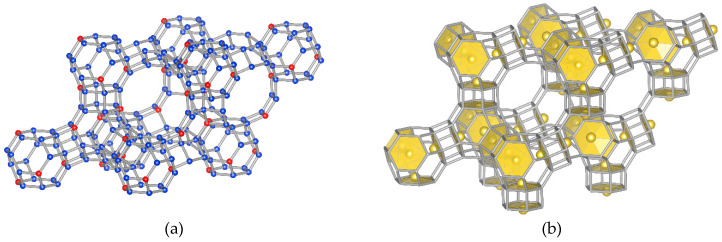

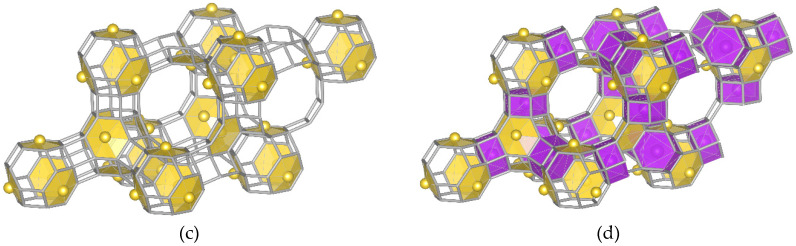
Fragment of the FAU structure (Me_32_Si_160_Al_32_O_384_, Fm-3c space group): (**a**) silicate FAU framework, (**b**) model “A”, (**c**) model “B” and (**d**) model “C” (descriptions of individual models in the text).

The chemical compositions of Me_32_Si_160_Al_32_O_384_ (Me = Na^+^, K^+^, Ag^+^) and Me_16_Si_160_Al_32_O_384_ (Me = Ni^2+^, Cu^2+^, Zn^2+^, Cd^2+^, Pb^2+^) were considered in order to reproduce the experimental Si/Al ratio equal to five for the investigated Me-Y samples [22]. The initial silicalite FAU framework was modified by substituting one silicon cation in each six ring in the unit cell (Figure 1a). At low occupancy (Si:Al > 2), cations are known to occupy sites I, I′ and II only [16]. Sites III and III′ are more or less unstable because of their weak interaction with the zeolite framework [18]. Therefore, three different models were prepared, depending on the extra-framework cations distribution:

- Model “A”—32 Me^+^ (or 16 Me^2+^ cations with 0.5 occ. in each position) in SI′ sites (Wyckoff pos. 32e) in the sodalite cage in front of the six-ring window connected to the hexagonal prism (Figure 1b),
- Model “B”—32 Me^+^ (or 16 Me^2+^) cations in SII sites (Wyckoff pos. 32e) in the 12-ring windows of the supercages (Figure 1c) and
- Model “C”—16 Me^+^ (or 8 Me^2+^) cations in SI sites (Wyckoff pos. 16c) located in the hexagonal prism connecting two sodalite cages and 16 Me^+^ (or 8 Me^2+^) cations in SII sites (Figure 1d).

### 2.1. Na-Y Structure Optimization

In order to select the best sodium distribution in the “C” model structure (model presented in Figure 1d), a series of calculations was carried out for various sodium distributions among (occupancy of) the I, I′ and II sites (Wyckoff pos. 16c, 32e and 32e, respectively). It has to be noted that sites I and I′ cannot be occupied simultaneously in the same hexagonal prism, because the Me–Me distance would be too small. The obtained results showed (Table 1) that the lowest total energy was obtained in the case of the last model (I + 0.5 × II), so in the following calculations for various extra-framework cations, this model was used. For each structure, the IR spectra were simulated and compared with the experimental ones and their properties analyzed in detail.

**Figure 2:**
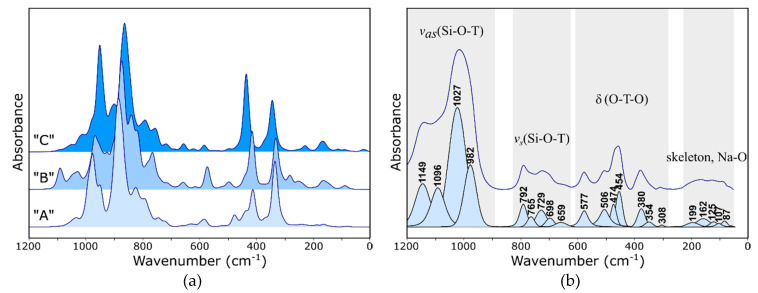
IR spectra of FAU: (**a**) calculated for the Na form of the “A”, “B” and “C” models (**b**) measured for the Na-Y zeolite.

Figure 2a shows the computed spectra of the “A”, “B” and “C” models presented in Figure 1b–d. Although it can be generally stated that the envelopes of all three spectra are similar, a detailed analysis of the intensity ratios of the individual bands shows some significant differences.

It should be noted that the compared models are based on an identical aluminosilicate framework and differ only in the location of the extra-framework cations. These cations interact with the skeleton and introduce different deformations. The spectral envelopes must, therefore, differ slightly. Apart from the analysis of the most intense bands related to the antisymmetric stretching and bending vibrations of Si–O–(Si,Al) bonds (in 1100–700 and 500–300 cm^−1^, respectively) and focusing on the pseudo-lattice range of the spectrum, it is necessary to take a closer look at the bands occurring in the range of 700–500 and <300 cm^−1^.

When analyzing the literature, one can come to the conclusion that, very often, the interpretation of zeolite spectra is based on determining the position of the so-called characteristic bands [3,23,24,25,26]. Due to the fact that most secondary building units are single or double rings of varying degrees, they are mostly ring vibrations. Characteristic ring vibrations are vibrations of the RO type (ring opening), which, by definition, cause simultaneous stretching and/or bending of Si–O–(Si,Al) bonds in phase with respect to the main axis ring [23]. In the case of double rings, PO (pore opening)-type vibrations were also selected, which caused the simultaneous and consistent phase shift of atoms in relation to the center of symmetry of the unit [3]. Visualizations of normal vibrations obtained on the basis of the presented calculations indicate that the bands at 698 and 577 cm^−1^ in the experimental spectrum of zeolite Na-Y (Figure 2b) should be considered as RO-type vibrations bands.

The second spectral range, particularly sensitive to the ion exchange process, is the range <300 cm^−1^. The model spectra (Figure 2a) have a completely different course here. This is not surprising, considering that many authors believe [27] that the bands in this range can be assigned to a specific cationic position. In the case of the “A” model, there are practically no active vibrations in the lattice region. When the SI′ site is fully occupied, the Me^+^ cations form a tetrahedron; we suspect that, during the vibration of such a system, the resultant dipole moment does not change (or changes very little), and the vibrations are slightly active in IR. Therefore, it seems that the SI and SII sites will be the most sensitive to ion exchange. Based on the visualization of normal vibrations in the model structures, bands in the experimental spectrum of the synthetic Y zeolite were assigned. The individual groups of bands are shown in Figure 2b. This interpretation agrees well with the literature data [5].

### 2.2. Model Selection for Various Cationic Forms

Similar considerations regarding the selection of the optimal cation distribution in the structure of faujasite were carried out for different cationic models. The range of calculations was narrowed down to the indicated the “A”, “B” and “C” models. The total energies calculated for the model FAU structures are collected in Table 2.

In the case of monovalent cations (Na^+^, K^+^ and Ag^+^), the model “C” is clearly the most energetically beneficial. This is confirmed in the related literature. In some works, faujasite was studied in situ in its dehydrated form [27,28]—it was found that the Na content was distributed equally over SI′ and SII. The distribution of cations between the SI and SI′ positions does not appear to follow the established rule, except that, in dehydrated samples, SI is favored over SI′ because of its octagonal symmetry [13]. Cations surrounded symmetrically with oxygen have a greater electrostatic stability than those bound to only three oxygen atoms in the less favorable C_3v_ symmetry. In hydrated samples, the opposite situation prevails, because the cations are then additionally coordinated by oxygen from water molecules.

In the case of divalent cations, SII turned out to be the most favorable position in the structure of faujasite. This is related to the minimization of electrostatic repulsion between the cations occupying this site [13]. At the same time, it should be noted that the differences between model B and model A were very slight in each case, which may indicate position SI′ as equally probable.

### 2.3. Influence of the Cation Guest on the Vibrational Spectra of Zeolite Y

**Figure 3:**
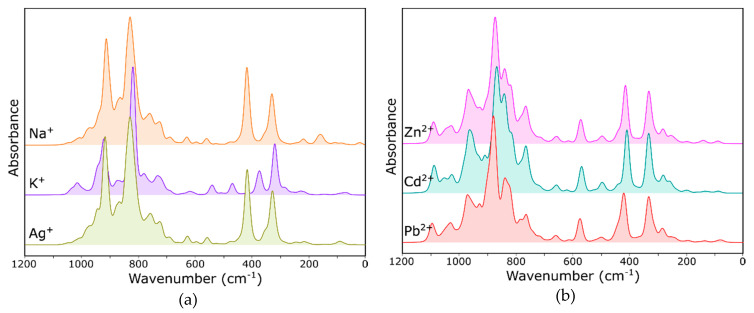
IR spectra of FAU: (**a**) calculated for exemplary monovalent cations and the “C” model and (**b**) calculated for exemplary divalent cations and the “B” model.

Taking into account the values of total energy summarized in Table 2, for further analysis, the IR spectra of model “C” and model “B”, calculated for monovalent and divalent cations, respectively, were selected. The exemplary spectra are shown in Figure 3. The theoretical spectra of all three model structures and eight different exchangeable cations (Na^+^, K^+^, Ag^+^, Ni^2+^, Cu^2+^, Zn^2+^, Cd^2+^ and Pb^2+^) are attached as Supplementary Figures S1–S3. Since the spectra calculated using all three models are very similar to each other and to the experimental ones, one can safely assume that, in real structures, extra-framework cations will be distributed statistically (but with a different probability [13]) among all three positions (I′, II and I). On the other hand, changing the cation site led to a significant deformation of the optimized structure and, thus, to changes in the theoretical IR spectral envelope.

The structure of zeolite should be considered as a set of interconnected (TO_4_) units ((SiO_4_) and (AlO_4_) tetrahedra). The strongest absorption bands of silicates lie in the wavenumber ranges 1200–950 (asymmetric stretching vibrations Si–O–Si and Si–O–Al) and 550–400 cm^−1^ (bending vibrations O–Si–O). In the 800–600 cm^−1^ region, there are bands corresponding to the symmetrical stretching vibrations Si–O–(Si,Al). The absorption spectra of the tectoaluminosilicates reflect the Al/Si order degree. Due to the orderly way of replacing silicon with aluminum, the bands in the model spectra are clearly separated. In the actual structure, a disorder in the replacement of Si by Al should be considered. This is revealed by the widening and flattening of the bands in these ranges. Additionally, in comparison with the model spectrum, additional bands related to the presence of terminal oxygen must appear in the experimental spectrum (the strength constants of the Si–O_b_ and SiO_t_ bonds differ).

**Figure 4:**
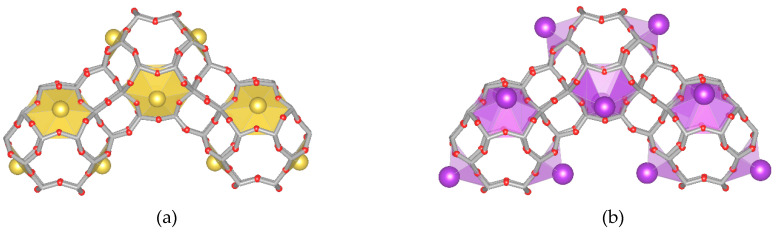
Fragment of the FAU structure with the M_32_Si_160_Al_32_O_384_ formula (model “B”), where M^+^: (**a**) Na^+^ in the SII sites and (**b**) K^+^ in the SII sites.

The deviations of individual O–T–O angles from the ideal value are very small. Therefore, no changes in the experimental spectra in the field of the vibration of T–O–(T) bonds should be expected. Therefore, the spectra of individual model structures are similar to each other. As we concluded in one of our previous papers [29], the theoretical spectra in the region relating to asymmetric stretching vibrations show only relatively small differences due to the similar interactions of these cations with all of the neighboring oxygen atoms. Only the theoretical spectra of the structure with cation guests with a large ion radius have a slightly different course. This is especially true for the K^+^ ions, while the spectra of the Na- and A- forms are almost identical (Figure 3a). The comparison of the K^+^ and Na^+^ sites is shown in Figure 4. The greater distance from the oxygen causes these potassium cations to interact much less with the skeleton, and thus, the degree of deformation of individual elements of the structure is smaller. This observation may indicate that the ionic radius, rather than the chemical nature, will have a dominant effect on the spectral envelope. The spectra of FAU structure with divalent ions (Figure 3b) are similar, because their ionic radii are also similar.

As mentioned, many studies indicate differences in the course of the far infrared spectra due to the different positions of the cations. Comparing the spectra of the FAU structures differing in a monovalent cation (Figure 3a), the differences in this range consist in the changes of both the integral intensity of the bands and their positions. In the case of divalent ions (Figure 3b), regardless of the type of cation, the envelope of the spectrum in this range is similar.

**Figure 5:**
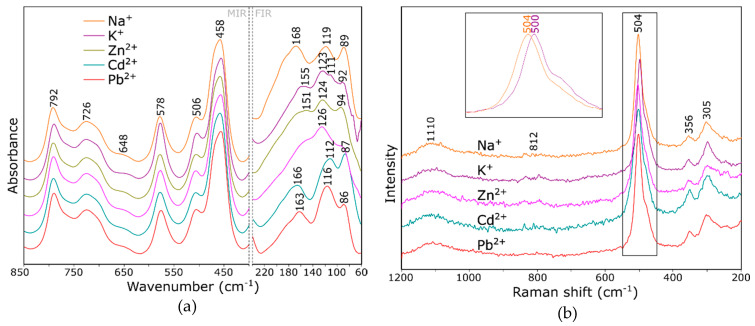
Fourier-transform infrared (FTIR) (**a**) and Raman (**b**) spectra of zeolite Y, with zeolite spectra with various cation guests.

Based on the presented computational spectra, an attempt was made to interpret the changes in the experimental ones (Figure 5). In our previous works [5,30], we suggested that ion exchange results in changes mainly of the intensity of the bands associated with RO vibrations. Positions of the bands in this range change to little a degree. Differences in the spectra are small but visible.

In the infrared spectrum (Figure 5a), the band at about 578 cm^−1^ seems to be the most sensitive to cation exchange. As can be concluded from the model spectrum (the band at about 450 cm^−1^ depending on the model; Figure 3), the intensity of this band is the highest for the ion with the largest radius (in the case of the analyzed experiment, these are K^+^ ions). The far-infrared range should be understood somewhat differently. The spectra in this range differ from the theoretical model due to the hydrated form of the sample zeolites—the degree of hydration will have a significant impact on the position of the cation in the zeolite structure [27].

It is worth noting that ring vibrations, due to their high symmetry, should be active mainly in Raman spectra. Although the physics of the phenomena underlying the use of these techniques is different, the information obtained through measurements is complementary.

In the GULP program, with a core shell model implemented, the ionic polarizability α is determined approximately in terms of the displacement of a massless shell with a charge X (representing valence electrons) from a core with all of the atom mass and charge Z (representing the nucleus and core electrons—i.e., electrons occupying inner shells), which are coupled by harmonic spring with force constant *k*. As a result, the calculated Raman spectra obtained in this study are not entirely reliable; therefore, it was decided not to subject them to deeper interpretation; however, they are included as Supplementary Figures S4–S6. The measured Raman spectra of the ion exchanged zeolite Y are shown in Figure 5b. The described calculation results confirmed [30] that the most intense bands in the Raman spectrum (at 504, 356 and 305 cm^−1^) are derived from the vibration of six-membered rings. Some other authors suggested [31] the dependence of the position of the mentioned bands on the degree of zeolite hydration but, also, on the related position of the cations in the zeolite framework. The observed changes concern both the change of position and the intensity ratios of the mentioned bands.

**Figure 6:**
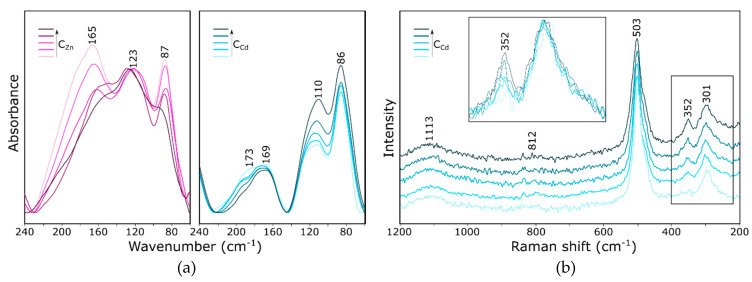
FTIR (**a**) and Raman (**b**) spectra of zeolite Y with varying degrees of Na^+^ by Zn^2+^ or Cd^2+^ substitution.

These changes are even more pronounced in the case of the gradual substitution of sodium by other ions. For example, in Figure 6, the vibrational spectra (both FTIR and Raman) as a function of the concentration of the solution introducing cadmium/zinc ions are shown. The differences in the infrared spectra of both exemplary cations are especially visible in the far infrared (Figure 6a). In the presented range, the intensity of the band at higher wavenumbers decreases (at 165 and 173 cm^−1^, respectively, for Zn^2+^ and Cd^2+^ sorption) towards the band at about 110 cm^−1^, which indicates a change in the position of the cations from SII to SI′ [27]; however, the validity of such an assignment has been questioned in a number of works [32,33]. The results of the calculations demonstrated that all three models and each cationic form had a slightly different spectrum in this range (Figures S1–S6). Therefore, it is not possible to precisely assign bands in the indicated range. The authors of various works [32,33] agreed that the cation vibrations participated in the infrared spectral range below 250 cm^−1^ for all cation sites and coupled with the low-frequency framework motions. Although the interpretation of the spectra based on specific positions of cations [27] seems correct, or at least probable, it is certainly very simplified (complete occupation of cationic positions, analysis of zeolites after dehydration and interpretation of only a part of the spectrum, without reference to the whole). Meanwhile, the exchange of ions of different sizes certainly causes some deformation of the framework. Based on the theoretical results obtained in our other studies (for zeolite A [34] and sodalite [35]), it can be concluded that the bands in this range should be associated with the same type of vibration, regardless of the type of cation. The position of the band shifts towards lower wavenumbers and the integral intensity increase with the increasing cation mass.

In the Raman spectrum (Figure 6b), a simultaneous increase in the integral intensity of the band at 352 cm^−1^ and the amount of cadmium in the zeolite structure is observed.

The theoretical models presented in this work are based on certain overly simplified theoretical and structural assumptions, such as: the classical lattice dynamics approach (accurate ab initio and DFT quantum mechanical approach is still prohibiting in terms of computer resources) and uniform distribution of aluminum in the structure (one aluminum atom for every five silicon ones in each six-membered ring), while, in a real structure, some statistical fluctuations in Si and Al atom distributions in the aluminosilicate framework (various Si/Al rations in various parts of the framework) are expected (especially when divalent extra-framework cations occupying the respective Wyckoff positions only partially are introduced); only selected models for extra-framework cation positions and distributions are studied, while such cations are most probably distributed among various positions statistically, with occupations changing from place-to-place in the structure, as well as the omission of the so-called zeolite water in the structure. It would be preferable to remove such assumptions one-by-one and to build more realistic models, but this is a very complicated and tedious task, requiring significant computer resources. Many such problems are not solved at the moment and left for future analyses.

Prospects for future research include attempts to employ accurate QM calculations with the total electronic charge distribution and topological analysis of bonding properties in order to determine bond orders and strengths, as well as calculations of changes in the bonding energy of cations within the aluminosilicate zeolite framework while comparing alkali cations (Na^+^ and K^+^) and heavy metal cations. At the same time, model studies may provide an answer to the question of why the structures of zeolites (in this case, faujasite) so effectively immobilize heavy metal cations. Heavy metals should bond more strongly with the skeleton, which may be responsible for the effectiveness of their immobilization and a large share of chemisorption in the discussed process.

## 3. Materials, Models and Methods

### 3.1. Computational Details

The calculations were carried out within the classical Lattice Dynamics approximation using the GULP [21] (General Utility Lattice Program) code. The atomic interactions are described by the Coulomb-Buckingham potential, and harmonic three-body terms were added for the O_Shell_–Si_Core_–O_Shel_ and O_Shel_–Al_Core_–O_Shell_ intra-tetrahedral angles to ensure the flexibility of the FAU framework (1).

(1) $$V\left(r_{ij}\right)=\frac{q_{i}q_{j}}{r_{ij}}+A_{ij}e^{-r_{ij}/\rho_{ij}}-\frac{C_{ij}}{r_{ij}^{6}}+\frac{1}{2}k_{3b}{\left(\theta -\theta_{0}\right)}^{2},$$

In Equation (1), the first term corresponds to the Coulomb potential, second and third define the Buckingham short-range potential and the last term describes the harmonic three-body potential. The symbols *i* and *j* refer to interacting ions; *q_i_* and *q_j_* to their charges (formal charges were used in this work); *A_ij_*, *ρij* and *C_ij_* are short-range Buckingham potential parameters; *k* is the bond force constant and *θ* the equilibrium bond angle. Ionic polarizability is modeled using the Dick-Overhauser shell model [36], in which an ion is represented by a separate core (representing the nucleus and inner shell electrons with the whole mass of the ion associated with it) and massless shell (mimicking the valence electrons), coupled by a harmonic spring with the force constant *k_c_* and the core shell potential, defined as (2).

(2) $$V\left(r_{ij}\right)=\frac{1}{2}k_{CS}r^{2}+\frac{1}{24}k_{CS4}r^{4},$$

*k_CS_*_4_ = 0 was assumed in this work. The polarizability of the isolated ion is proportional to an ion charge, $q_{s}^{2}$; the sum of the core and shell charges is equal to the formal charge of the ion. In this work, the shell model is used for oxygen anion and nickel and copper cations only. IR and Raman intensities are calculated in the GULP Program in an approximate way, i.e., IR intensities are computed using the Born effective charge tensor, while Raman intensities are calculated using the derivatives (with respect to the atomic displacements) of the Raman susceptibilities χ = π/4 * (ε^∞^ − δ_ab_), where ε^∞^ is the high frequency dielectric constant tensor, and δ_ab_ is the delta function that is one for xx, yy, zz and, otherwise, 0. The optimized parameters of potentials used in the calculations are presented in Table 3, Table 4 and Table 5 (r_min_ and r_max_ in Table 3 define the range over which the two-body interatomic potential acts, while the cutoffs in Table 4 are values defining the range of potential acting between atoms 1-2, 1-3 and 2-3, respectively, analogously as r_max_ in the case of two-body potential). For all model structures, full geometry optimization (unit cell and atomic positions) was done, and for the resulting relaxed structures, Hessian eigenvalues and IR intensities were calculated. The IR spectra were simulated by a broadening procedure as the 1:1 linear combination of the Gaussian and Lorentzian analytical functions with FWHM = 20 cm^−1^ and frequency and height corresponding to the respective eigen frequencies of the active IR modes.

### 3.2. Experimental

In the experimental part of this work, sodium forms of zeolite Y (Zeolyst International, Conshohocken, PA, USA) were used as the starting material. Sorption of various cations on the sodium forms of zeolite Y was studied using aqueous solutions of metal nitrates. A suspension of the zeolite in water (20 g/dm^3^) was shaken with the appropriate metal salt solution for 24 h at 80 °C and centrifuged. After the ion exchange process, the samples were triply washed with distilled water and then dried at 80 °C for several days. Phase compositions of studied materials were confirmed using X-ray diffraction (XRD) (results not presented here). Infrared spectra of different forms of zeolites were measured using a Bruker VERTEX 70v vacuum spectrometer (Bruker, Billerica, MA, USA). They were collected in the region of 4000–60 cm^−1^ after 256 scans at 2-cm^−1^ resolution using the standard KBr and polyethylene pellet methods for MIR and FIR spectra, respectively. The Raman spectra were measured on a Bruker RamanScope III FT-Raman spectrometer (Bruker, Billerica, MA, USA), coupled with an Olympus optical microscope (Shinjuku, Tokio, Japonia). Raman spectra were collected in the region of 4000–100 cm^−1^ after 256 scans at 2-cm^−1^ resolution. All spectra were corrected with a linear baseline.

## 4. Conclusions

One of the important goals of the work was to use the results obtained from the calculations carried out for model FAU structures and the theoretical spectra resulting from these calculations for a rather complicated interpretation process of experimental faujasite vibrational spectra. Good concordance of the theoretical and real spectra allows for inference about the correctness of the model structure and the correctness of the assigned bands on the experimental spectra. We anticipate that the calculated infrared and Raman spectra presented here will be useful in future experimental characterizations of ion-exchanged zeolites, regardless of the final application.

Based on the results obtained, the following conclusions can be drawn:

- Theoretical spectra are a useful tool for the interpretation of experimental ones, allowing the assignment of bands to individual normal modes of vibrations.
- Bands changing both the intensity and position, depending on the type of extra-framework cation in the structure, were identified both in the IR and Raman spectra of faujasite. Based on the literature data, these bands were assigned to fully symmetrical vibrations of six-membered rings—the so-called ring opening (RO) vibrations.
- In far IR, one can observe significant differences between the theoretical spectra, which results from different characters of respective interactions between extra-framework cations and the host aluminosilicate framework.
- MIR range spectra for the Y zeolite measured after the sorption process shows visible changes in the pseudo-lattice vibrations caused by the ion exchange process.
- In the IR spectrum, the band located at approx. 578 cm^−1^ can be assigned to bending the vibrations of Si–O–Al bridges; the position and intensity of this band changes significantly with a type of nontetrahedral cation and its position relative to the six-membered ring.
- The Raman spectrum, as more sensitive to changes in the symmetry of the system, is a good indicator of the sorption process. The bands at 503 and 352 cm^−1^ undergo changes in position and integral intensity, respectively.
- Similar observations can be made also in the case of spectra obtained for all analyzed “A”, “B” and “C” model structures. Since the spectra calculated using all three models are very similar to each other and to the experimental ones, one can safely assume that, in real structures, nontetrahedral cations will be distributed statistically (but with different probabilities) among all three positions (II, I′ and I).

## Figures and Tables

**Table 1:** Total energies calculated for the Na_32_-Y structure with various sodium spatial distributions.

Model Structure	Total Energy (eV)
0.5 × I′ + 0.5 × II	−5868.37
0.666 × I + 0.666 × II	−5869.32
0.666 × I′ + 0.333 × II	−5869.84
0.333 × I′ + 0.666 × II	−5870.07
I + 0.5 × II	−5873.83

**Table 2:** Total energies calculated for the model FAU structures.

Total Energy (eV)			
Cation	Model “A”	Model “B”	Model “C”
Na^+^	−5920.48	−5921.07	−5937.11
K^+^	−5897.30	−5906.96	−5916.51
Ag^+^	−5921.83	−5922.54	−5940.92
Ni^2+^	−5926.12	−5926.55	−5915.52
Cu^2+^	−5927.76	−5928.13	−5918.72
Zn^2+^	−5919.86	−5920.30	−5910.80
Cd^2+^	−5915.26	−5916.41	−5908.17
Pb^2+^	−5928.04	−5928.41	−5918.63

**Table 3:** Parameters of the Buckingham potentials used in this work.

Interaction	*A* (eV)	*ρ* (Å)	*C* (eVÅ^6^)	r_min_ (Å)	r_max_ (Å)
O_S_-O_S_	22,764.3	0.149	27.879	0	12
Si_C_-O_S_	1283.907	0.32052	10.66158	0	10
Al_C_-O_S_	1460.3	0.29912	10	0	10
Na_C_-O_S_	280.1	0.35771	10	0	10
K_C_-O_S_	1000.3	0.36198	10.569	0	10
Ag_C_-O_S_	1417.03	0.2851	10	0	10
Ag_C_-Ag_C_	1335.89	0.1424	10	0	10
Ni_S_-O_S_	1582.5	0.2882	10	0	10
Cu_S_-O_S_	5950.2	0.2427	10	0	12
Zn_C_-O_S_	539.7	0.3581	10	0	10
Cd_C_-O_S_	868.3	0.35	10	0	10
Pb_C_-O_S_	2950.4	0.261	10	0	10

**Table 4:** Parameters of the three-body harmonic potentials used in this work.

Interaction	*k*_3*b*_ (eV rad^−2^)	*θ*_0_ (deg)	Cutoffs (Å)		
O_S_-Si_C_-O_S_	2.09724	109.47	1.9	1.9	3.5
O_S_-Al_C_-O_S_	2.09724	109.47	1.9	1.9	3.5

**Table 5:** Parameters of the core–shell harmonic potentials used in this work.

Species	Core Charge	Shell Charge	*k_CS_* (eVÅ^−2^)	*k_CS_*_4_ (eVÅ^−4^)
Al	3	-	-	-
Si	4	-	-	-
O	0.86902	−2.86902	74.92	0
Na	1	-	-	-
K	1	-	-	-
Ag	1	-	-	-
Cu	1	1	100	0
Ni	−1.344	3.344	50	0
Zn	2	-	-	-
Cd	2	-	-	-
Pb	2	-	-	-

## Data Availability

Data presented in this study are available on request from the corresponding author.

## Supplementary Materials

The following are available online. Figure S1: IR spectra of FAU calculated for the “A” model. Figure S2: IR spectra of FAU calculated for the “B” model. Figure S3: IR spectra of FAU calculated for the “C” model. Figure S4: Raman spectra of FAU calculated for the “A” model. Figure S5: Raman spectra of FAU calculated for the “B” model. Figure S6: Raman spectra of FAU calculated for the “C” model.

## Sample Availability

Samples of the compounds are available from the authors.
