# Supplementary material for: Vibrational Spectra of Zeolite Y as a Function of Ion Exchange
Source: Molecules. 2021 Jan 11;26(2):342. doi: 10.3390/molecules26020342 (PMC7827790; doi:10.3390/molecules26020342)
Supplement: Supplementary file 1 [file molecules-26-00342-s001.pdf]

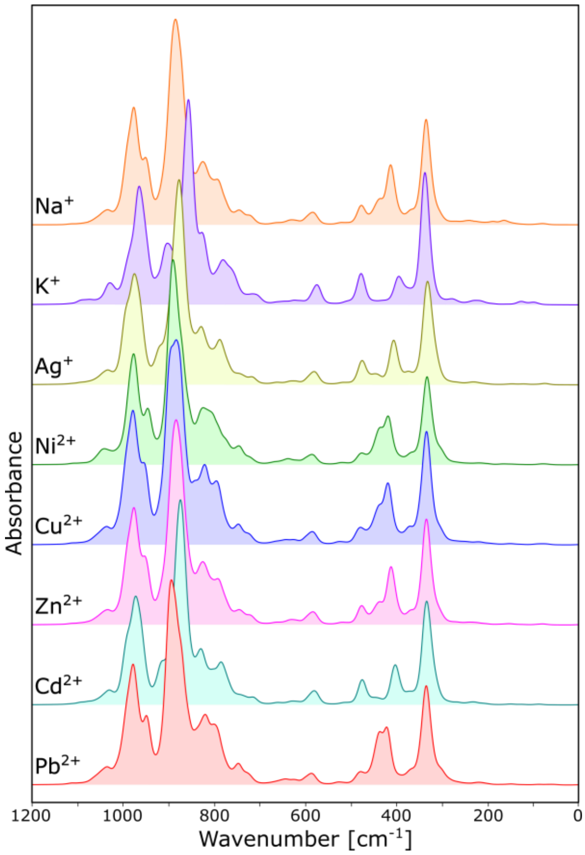

Figure S1. IR spectra of FAU calculated for "A" model

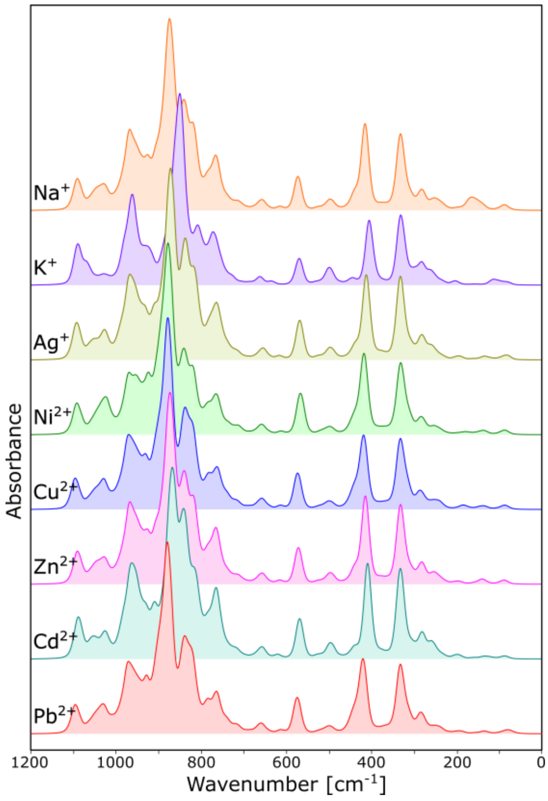

Figure S2. IR spectra of FAU calculated for "B" model

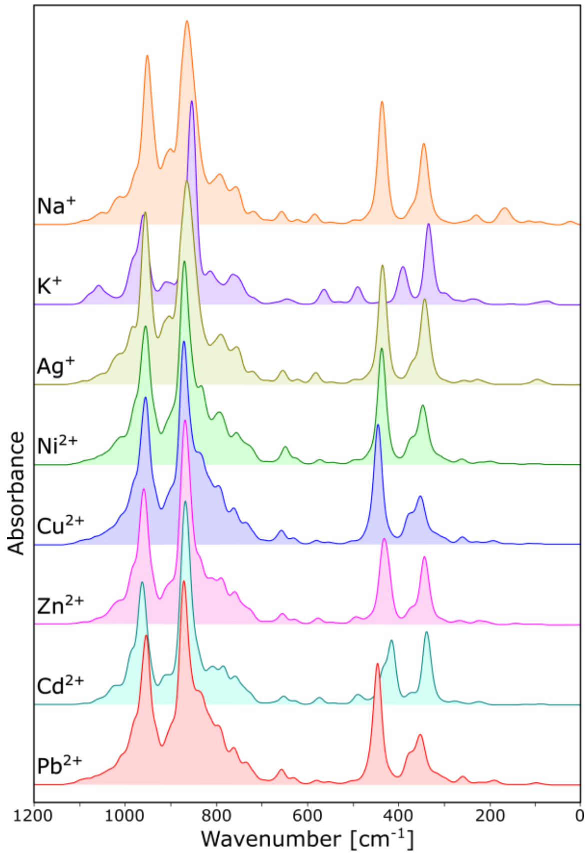

Figure S3. IR spectra of FAU calculated for “C” model

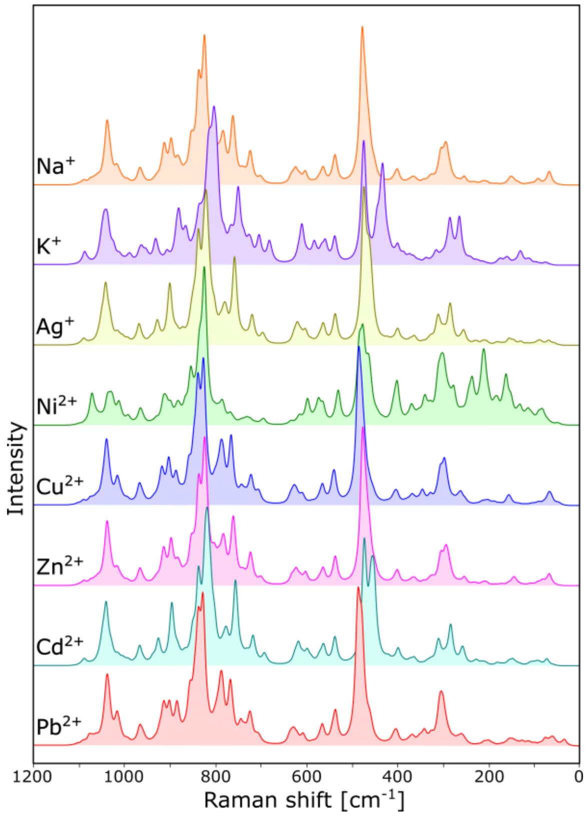

Figure S4. Raman spectra of FAU calculated for “A” model

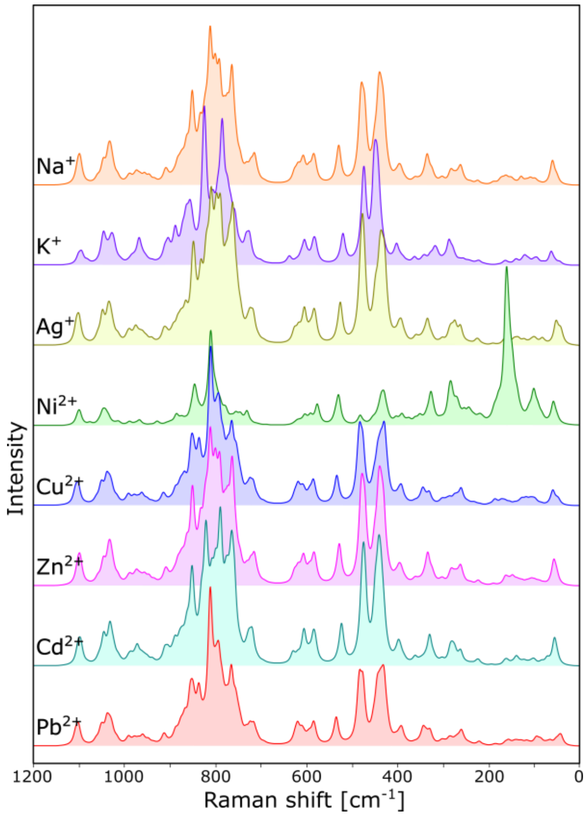

Figure S5. Raman spectra of FAU calculated for "B" model

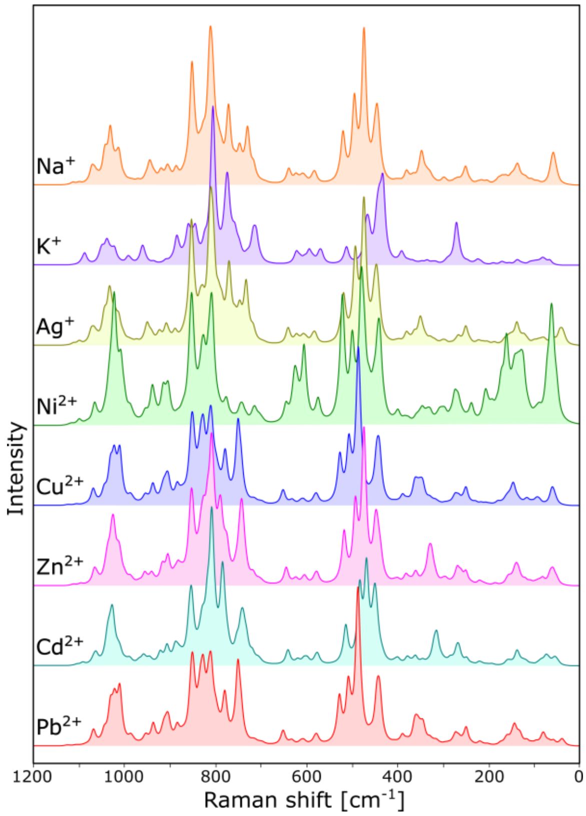

Figure S6. Raman spectra of FAU calculated for "C" model
